# Isolation and Biophysical Characterisation of Bioactive Polysaccharides from Cucurbita Moschata (Butternut Squash)

**DOI:** 10.3390/polym12081650

**Published:** 2020-07-24

**Authors:** Shahwar Imran Jiwani, Richard B. Gillis, David Besong, Fahad Almutairi, Tayyibe Erten, M. Samil Kök, Stephen E. Harding, Berit S. Paulsen, Gary G. Adams

**Affiliations:** 1 Queen’s Medical Centre, Faculty of Medicine and Health Sciences, University of Nottingham, Clifton Boulevard, Nottingham NG7 2UH, UK; ntzrbg@exmail.nottingham.ac.uk; 2 Solar and Photovoltaics Engineering Center, King Abdullah University of Science and Technology, Thuwal, Makkah 23955-6900, Saudi Arabia; david.tabotmorou@kaust.edu.sa; 3 Department of Biochemistry, Faculty of Science, University of Tabuk, P.O. Box 741, Tabuk 71491, Saudi Arabia; falrabae@ut.edu.sa; 4 Department of Nutrition and Dietetics, Faculty of Health Sciences, Bayburt University, 69000 Bayburt, Turkey; tayyibeerten@bayburt.edu.tr; 5 Department of Food Engineering, Faculty of Engineering & Architecture, Abant Izzet Baysal University, Gölköy, 14300 Bolu, Turkey; kok_s@ibu.edu.tr; 6 National Centre for Macromolecular Hydrodynamics (NCMH), School of Biosciences, Sutton Bonington Campus, The University of Nottingham, Sutton Bonington, Leicestershire LE12 5RD, UK; steve.harding@nottingham.ac.uk; 7 Department of Pharmaceutical Chemistry, School of Pharmacy, Section Pharmacognosy, University of Oslo, PB 1068, Blindern, N-0316 Oslo, Norway; b.s.paulsen@farmasi.uio.no

**Keywords:** *Cucurbita moschata*, GCMS, levan, pectin, bioactivity, hydrodynamics

## Abstract

Cucurbits are plants that have been used frequently as functional foods. This study includes the extraction, isolation, and characterisation of the mesocarp polysaccharide of *Cucurbita moschata*. The polysaccharide component was purified by gel filtration into three fractions (NJBTF1, NJBTF2, and NJBTF3) of different molecular weights. Characterisation includes the hydrodynamic properties, identification of monosaccharide composition, and bioactivity. Sedimentation velocity also indicated the presence of small amounts of additional discrete higher molecular weight components even after fractionation. Sedimentation equilibrium revealed respective weight average molecular weights of 90, 31, and 19 kDa, with the higher fractions (NJBTF1 and NJBTF2) indicating a tendency to self-associate. Based on the limited amount of data (combinations of 3 sets of viscosity and sedimentation data corresponding to the 3 fractions), HYDFIT indicates an extended, semi-flexible coil conformation. Of all the fractions obtained, NJBTF1 showed the highest bioactivity. All fractions contained galacturonic acid and variable amounts of neutral sugars. To probe further, the extent of glycosidic linkages in NJBTF1 was estimated using gas chromatography–mass spectrometry (GCMS), yielding a high galacturonic acid content (for pectin polysaccharide) and the presence of fructans—the first evidence of fructans (levan) in the mesocarp. Our understanding of the size and structural flexibility together with the high bioactivity suggests that the polysaccharide obtained from *C. moschata* has the potential to be developed into a therapeutic agent.

## 1. Introduction

Polysaccharides play crucial roles in the regulation of various biological processes [1]. They are also used as a gelling, stabilising, or thickening agent in food products [2,3]. In the pharmaceutical industry, polysaccharides are generally used as excipients during drug manufacture based on their biophysical properties, such as hydrophilic properties, emulsification, and viscoelasticity [4]. Naturally-extracted polysaccharides are a preferred alternative over synthetic polymers during the development of a controlled drug release system as well as food additives because of their biocompatibility, biodegradability, low toxicity, and low cost [5,6]. However, the complexity in constituents, and the isolation of the polysaccharide therein, proves to be a challenge in biomedical and nutraceutical applications [7,8], and this is true for the bioactive polysaccharides from *Curcurbita*.

*Cucurbita* is a genus of plant reported to be rich in complex polyuronides (pectins) composed chiefly of domains of homogalacturonan, rhamnogalacturonan-I, and -II [9]. The consumption of these dietary glycans has been shown to control glycaemic levels and to promote biological activity directly or by the activation of complex reaction cascades [10,11]. The higher complement activity is associated with the ramified “hairy (branched) region” of pectin compared to the smooth backbone [12].

Polysaccharides from one species of *Curcurbita,* namely *C. moschata*, is particularly promising with regards to control of the glycaemic index in humans. It is a medicinal plant with hypoglycaemic, hypolipidaemic [13,14], antitussive [15], and immune-stimulating properties [16]. Despite this plethora of literature, emphasising the health significance of cucurbit polysaccharides and their solution structure (hydrodynamic behaviour), in addition to their full potential for inducing bioactivity, remains unexplored. The study aimed to isolate, fractionate, and probe the structural properties and bioactivity of polysaccharides extracted from *C. moschata.*

## 2. Methods

### 2.1. Isolation of Polysaccharide 

Butternut squash was purchased from a local market in Nottingham, UK. Extraction methodology of [17] was followed. The pulp from the fruit of *C. moschata* was chopped into pieces (1 cm^3^ approximately), dried, and ground to make a powder. The powdered pulp was dispersed in deionised water (1 g in 20 mL) at 45 °C and centrifuged at 4800 rpm for 25 min at 20 °C using a Beckman centrifuge Model J2-21M (Minneapolis, Minnesota, US). The supernatant was collected and concentrated in a rotary evaporator (BUCHI Rotavapor™ R-100 Rotary Evaporator, Fisher Scientific, Loughborough, UK) at 45 °C until the volume reduced to one fifth of the original volume. The concentrate was filtered using 11mm Whatman filter paper (Whatman, Maidstone, UK). The filtrate was washed twice with chilled 95% and absolute ethanol, respectively. Each washing was followed by centrifugation at 4800 rpm at 4° C for 25 min and the pellet collected and freeze-dried using freeze drier (Super Modulyo, ThermoSavant, Knutsford, UK). The freeze-dried pellet was dispersed in distilled water (1 mg/20 mL) and dialysed against deionised water using a dialysis membrane (BioDesignDialysis tubing D106, Fisher Scientific, Loughborough, UK) with 8000 molecular weight cut off for 72 h.

The dialysate was mixed and washed three times with Sevage reagent (butanol with chloroform in a 5:1 (*v*/*v* ratio) to remove proteins from the polysaccharides [18]. The washed sample was later mixed with absolute ethanol followed by centrifugation at 4800 rpm at 4 °C for 25 min. The pellet was collected and freeze-dried for 48 h.

For confirmation of the presence of polysaccharides, methodology of [19] for the total sugar test and uronic acid detection was adapted. The detection of protein was determined by the Biuret test [19].

### 2.2. Gel Chromatography

Sephacryl 400 column (2.1 × 50 cm^2^) was prepared using 0.1 M sodium acetate, 0.02 M EDTA pH 6.5 buffer. Next, 50 mg/mL of crude polysaccharide was loaded onto the column. Eluent was tested for the presence of polysaccharide using phenol-sulfuric acid assay. Polysaccharide-rich fractions were divided into three groups (NJBTF1, NJBTF2, and NJBTF3) and freeze-dried for further analysis.

### 2.3. Hydrodynamic Characterisation

#### 2.3.1. Sample Preparation

The stock solution was prepared by dissolving 100 mg of freeze-dried powder (of isolated and fractionated polysaccharide) in 10 mL of water followed by dialysis in 0.1 M of phosphate buffer saline (PBS: Na_2_HPO_4_.12H_2_O, KH_2_PO_4,_ NaCl), pH 7.0 (Green, 1933). The sample concentration was measured using an Atago DD-7 refractometer (Atago, Japan); dn/dc of 0.146 mL/g [20]. Stock solutions at 2.0 mg/mL were prepared for analysis of each. 

#### 2.3.2. Intrinsic Viscosity 

Relative viscosities of all samples in solution (relative to solvent) were measured using an Ostwald viscometer. The flow times were recorded at 20.00 ± 0.05 °C. From the solution/solvent flow-time ratio, the kinematic relative viscosity was obtained. Because of the low concentrations used, a correction for density effects was deemed negligible and dynamic relative viscosities were approximated as kinematic values: i.e., because of the low concentrations, a density correction was not necessary [21]. The intrinsic viscosity [η] was found by extrapolation to infinite dilution of the reduced and inherent viscosities plotted against concentration to eliminate the effects of non-ideality using the Huggins and Kraemer equations, respectively.

(1) $$\eta_{red}=\left[\eta \right]\left(1+K_{H}\left[\eta \right]c\right)$$

(2) (lnηrel)c=[η](1−KK[η]c)

where *c* is the concentration, $K_{H}$ and $K_{K}$ are the Huggins and Kraemer coefficients, repectively.

The Solomon-Ciuta equation was used as an additional check [21].

(3) [η]=[2(ηrel−1)−2ln(ηrel)]12c

#### 2.3.3. Sedimentation Velocity

A Beckman Optima XL-I analytical ultracentrifuge (Indianapolis, Indiana, USA) was used to perform sedimentation velocity experiments. Solutions were centrifuged at 40,000 rpm (~120,000 g) at a temperature of 20.0 ± 0.1 °C for 12 h. Data were analysed using the c(s) algorithm of Dam and Schuck [22], chosen over g(s) because of the low s-values (and poor resolution from the air/solvent meniscus).

Sedimentation coefficients, *s* (in Svedberg units, 1 S = 10^−13^ seconds), were corrected to standard solvent conditions (density and viscosity of water at 20.0 °C, van Holde, 1971), and then a plot of 1/*s*_20,w_ vs. c (corrected for radial dilution) was plotted and fitted to the Gralen equation

(1/*s*_20,w_) = (1/*s*^o^_20,w_) (1 + *k*_s_ c) (4)

where *k*_s_ is the Gralen coefficient, to yield the non-ideality corrected *s*^o^_20,w_ from the reciprocal of the intercept.

#### 2.3.4. Sedimentation Equilibrium

The Beckman Optima XL-I analytical ultracentrifuge was also used to perform sedimentation equilibrium experiments to obtain the weight average molecular weights *M*_w_ (in kDa). The loading amount of sample and reference solvent solutions was 80 µL. Solutions were centrifuged at 10,000 rpm (~8000 g) at a temperature of 20.0 ± 0.1 °C for 72 h. Data acquired from the experiment were analysed using the SEDFIT-MSTAR program (Bethesda, Rockville, MD, USA) [23]. A partial specific volume of 0.65 mL/g was used. To minimize the effects of thermodynamic non-ideality, the lowest possible loading concentration was employed (0.4 mg/mL), and results for the apparent point weight average molar masses *M*_w,app_(r) as functions of radial position *r* in the ultracentrifuge extrapolated to zero concentration *c*(*r*) = 0.

#### 2.3.5. *M*_SM_ Estimates

Estimates of the Scheraga-Mandelkern molecular weights, MSD, were also made from *s*_20,w_, and [*η*]. This approximate transport-based method is based on the relative invariance with the shape of a parameter β (2.1–2.5 for most conformations) and gives an average molar mass between that of the number and weight averages [21,24].

(5) *M*_SM_ = [(N_A_.*s*^o^_20,w_·[*η*]^1/3^·*η*_0_)/β(1 − ῡ*ρ*)·100^1/3^]^3/2^

where N_A_ is Avogadro’s number, *η*_ο_ is the viscosity of water at 20.0 °C, ῡ is the partial specific volume, and *ρ*_o_ the density of water at 20.0 °C. A value for β of (2.3 ± 0.2) × 10^6^ was taken.

#### 2.3.6. Conformational Flexibility

For an estimate of chain flexibility, we can use the persistence length, *L_p_*, which has theoretical limits of 0 for a random coil and ∞ for a stiff rod. Practically the limits are ~1–2 nm for a random coil and ~200–300 nm for a very stiff rod-shaped macromolecule. 

We use the MULTI-HYDFIT algorithm of Ortega and Garcia de la Torre (2007) [25,26] to estimate *L_p_*. The method combines the Bushin–Bohdanecky equation [27,28].

(6) $${\left(\frac{M_{w}^{2}}{\left[\eta \right]}\right)}^{1/3}=A_{0}M_{L}\phi^{-1/3}+B_{0}\phi^{-1/3}{\left(\frac{2L_{p}}{M_{L}}\right)}^{-1/2}M_{w}^{1/2}$$

where ϕ is the Flory-Fox coefficient (2.86 × 10^23^ mol^−1^) and *A*_0_ and *B*_0_ are tabulated coefficients, and the Yamakawa–Fujii equation [29]

(7) $$s20,w^{0}=\frac{\left(M_{L}-\bar{v}\rho_{0}\right)}{3\pi \eta_{0}N_{A}}\times \left[1.843{\left(\frac{M_{w}}{2M_{L}L_{p}}\right)}^{1/2}+A_{2}+A_{3}{\left(\frac{M_{w}}{2M_{L}L_{p}}\right)}^{-1/2}+\ldots \right]$$

where *A*_2_ is −*ln(d/2L_p_)*, with d as the chain diameter and *A*_3_ = 0.1382 [30]. MULTI-HYDFIT estimates the best range of values of *L_p_* and *M_L_* based on minimisation of a target function Δ. An estimate for the chain diameter, *d*, is also required but extensive simulations have shown that the results returned for *L_p_* are relatively insensitive to the value chosen for *d* which was fixed at an average of ∼0.8 nm [30,31].

### 2.4. Complement Fixation

Complement fixation assay was carried out to identify the bioactivity of the fractionated polysaccharide. Method B of Michaelson was followed [32].

Fractions (NJBTF1, NJBTF2, and NJBTF3) were incubated with human serum (the complement source) in order to identify their influence on the human complement system. It was expected that the addition of polysaccharide fractions could either inhibit or activate the complement factors. In both conditions (activation/inhibition), complement activity was depleted and inhibition of lysis occured. Inhibition of the lysis induced by the polysaccharide sample in the haemolysis inhibition system was used to measure the ICH_50_ (the concentration of the polysaccharide samples required to induce 50% lysis). BPII, a polysaccharide fraction from *Biophytum petersianum*, was used as a positive control. The inhibition of lysis was calculated using the formula [(A_control_ − A_test_)/A control] × 100% [12]. A plot of the inhibition of lysis (%) against the concentration (µg/mL) of sample was constructed to identify the concentration of the sample able to give 50% inhibition. 

### 2.5. Methanolysis for Monosaccharide Composition Determination Using Gas Chromatography

The monosaccharide compositions were determined by gas chromatography of the trimethylsilylated derivatives of the methyl glycosides obtained by methanolysis of the samples using 4 M HCl in anhydrous methanol at 80.0 °C for 24 h [33]. Instrument used was Thermo scientific focus GC with Resttek–Rxi 5MS (length: 30 m, diameter: 23 mm, thickness: 0.25µm) columns. Flow rate details are: Flow mode: pressure control, Flow value: 1.4 mL/min, Flow nominal: 0.01 mL/min. The instrument had flame ionization detector with H_2_ and splitt/splittles Injector (Split ratio 1:10). Helium gas (flow 0.70 bar with constant pressure) was used.

### 2.6. GCMS and Linkage Determination

Derivatisation of polysaccharide was carried out [34]. Analysis (of the partly acetylated, partly methylated alditols) was performed using 1 µL of aliquots by GC-MS (GC-8000 series instrument; detector: fission instruments, MD800; column: factor FOUR™, VF-1 ms, injection temperature: 250.0 °C) [12]. The compounds at each peak were characterised by an interpretation of the retention times and the characteristic mass spectra. The estimation of the relative amounts of each linkage type was related to the total amount of each monosaccharide type as determined by methanolysis.

## 3. Results and Discussion

### 3.1. Intrinsic Viscosities

**Figure 1:**
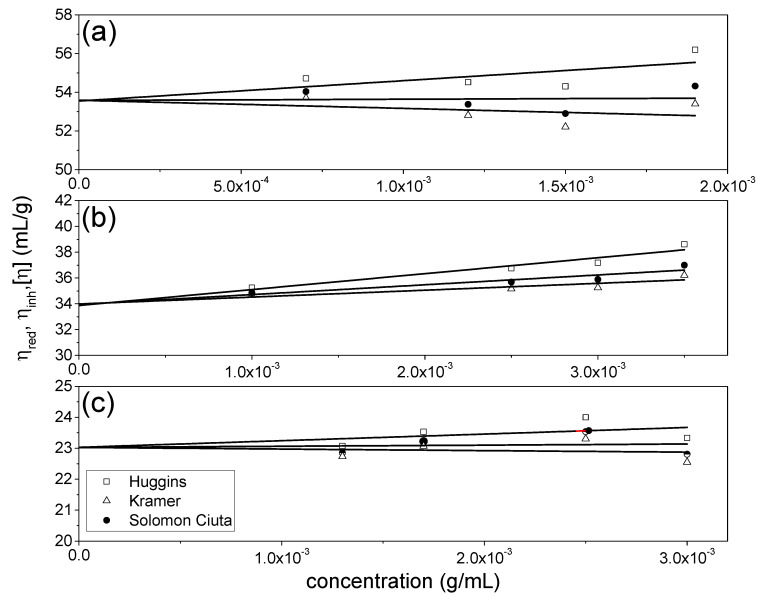
Intrinsic viscosity plots of *Curcurbita moschata* polysaccharides (**a**) NJBTF1, (**b**) NJBTF2, (**c**) NJBTF3. □: reduced viscosities, η_red_, fitted by the Huggins equation; ∆: inherent viscosities, η_inh_, fitted by the Kraemer equation; ●: Solomon-Ciuta estimates ([η]_sc_) with a linear extrapolation to c = 0. Solvent: phosphate chloride buffer (pH 7.0, I = 0.1 M), temperature = 20.0 °C.

The results for the NJBTF fractions are compared in Figure 1 and Table 1. Encouragingly, consistent results are obtained from the Huggins and Kraemer extrapolations and also from the Solomon-Ciuta equation, with values of ~54, 34, and 23 mL/g for NJBTF1, NJBTF2, and NJBTF3 respectively, typical for pectic types of polysaccharides although somewhat higher compared with those obtained from other preparations [35,36].

### 3.2. Sedimentation Coefficients and Distributions

**Figure 2:**
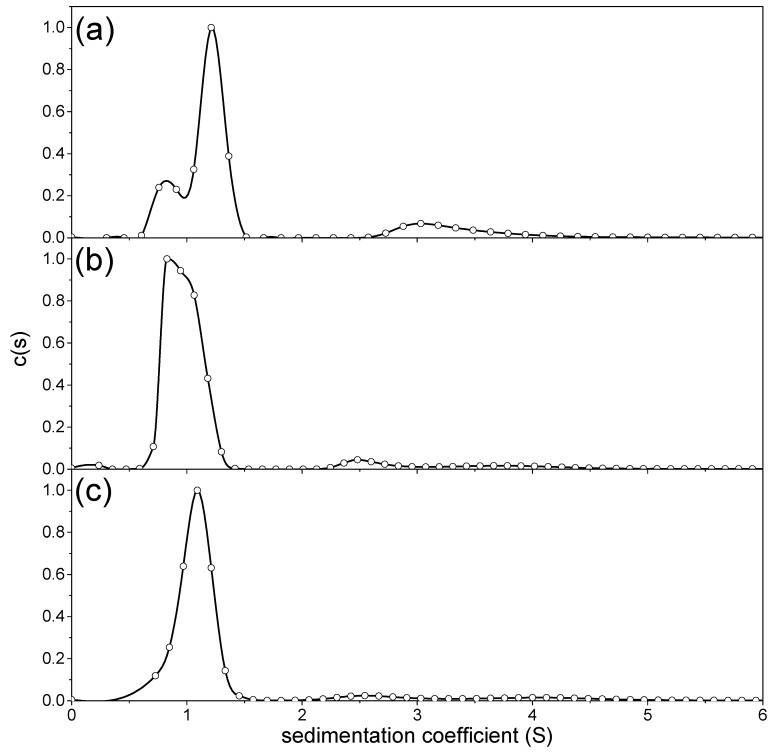
SEDFIT c(s) vs. s sedimentation coefficient distribution profiles obtained at 2 mg/mL (**a**) NJBTF1, (**b**) NJBTF 2, (**c**) NJBTF3. Maximum values for c(s) were normalised for comparison purposes. Solvent: phosphate chloride buffer (pH 7.0, I = 0.1 M), temperature = 20.0 °C.

The *c*(*s*) vs. *s* distributions are shown in Figure 2. It is clear that two (NJBTF1, 3) or three (NJBTF2) discrete components are seen in the fractions, a feature commonly seen in preparations of some classes of polysaccharide, even after fractionation [37].

Values of the weight average sedimentation coefficients for each component, *s*_20,w_ (normalised to standard solvent conditions, namely the density and viscosity of water at 20.0 °C), were obtained for each component from SEDFIT [38] and shown in Table 2, along with the relative proportions of each discrete component. These species were generally present at all concentrations and at the same weight percentage (Table 2).

**Figure 3:**
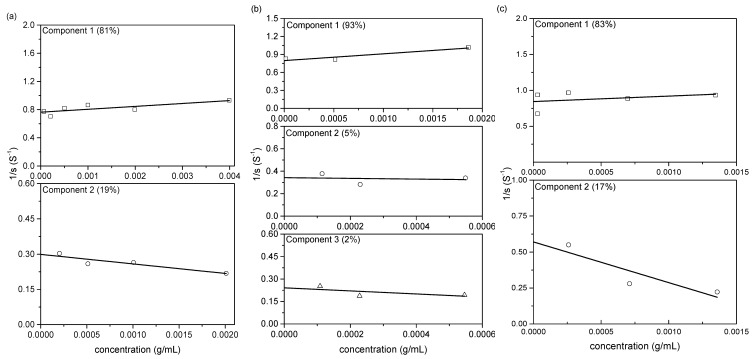
Concentration dependence of the reciprocal sedimentation coefficient (**a**) NJBTF1, (**b**) NJBTF2, (**c**) NJBTF3.

Extrapolation of the series of the reciprocal s_20,w_ values, obtained at a series of concentrations of the sample up to zero concentration, gave the weight average *s* ^0^_20,w_ for the polysaccharide (Figure 3).

### 3.3. Molecular Weights

**Figure 4:**
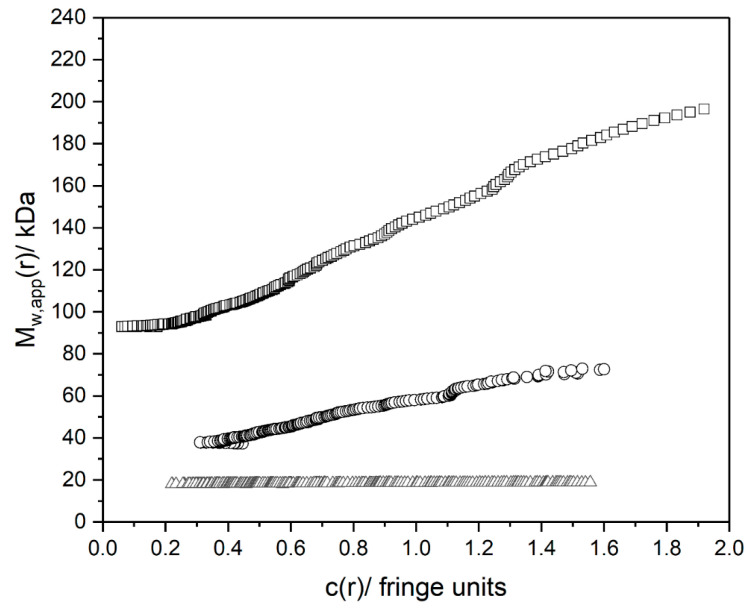
Sedimentation equilibrium analysis (using SEDFIT-MSTAR) on *C. moschata* polysaccharides. A low loading concentration (to minimise effects of thermodynamic non-ideality) of 0.4 mg/mL (~0.8 fringes in the long path-length cells) was employed. Plots of point weight average molecular weights *M*_w,app_(*r*) in kDa vs. local concentration *c*(*r*) (in Rayleigh fringe units) as functions of radial displacement, r, from the centre of rotation. Squares NJBTF1, Circles NJBTF2, Triangles NJBTF3. M_w_ values (M_w,app_) extrapolated to zero concentration = 92 ± 5 kDa (NJBTF1), 31 ± 2 kDa (NJBTF2), and 19 ± 1 kDa (NJBTF3). Solvent: phosphate chloride buffer (pH 7.0, I = 0.1 M), temperature = 20.0 °C.

Figure 4 shows the point weight average *M*_w,app_(*r*) vs. *c*(*r*) plots from SEDFIT-MSTAR for the three fractions. The lowest molecular weight fraction shows almost no change of *M*_w,app_ with concentration, but the higher one shows an increase with *c*(r), indicative of some degree of self-association.

Table 3 shows the values extrapolated to zero concentration and are in the range of 19–90 kDa, which are as expected from the corresponding intrinsic viscosity values (Table 1). This table also compares the approximate *M*_SM_ values obtained from the Scheraga-Mandelkern equation (Equation (5)) with *M*_w,app_. As expected for a polydisperse system, the *M*_w_ values from sedimentation equilibrium are > *M*_SM_ [39].

### 3.4. Persistence Length

**Figure 5:**
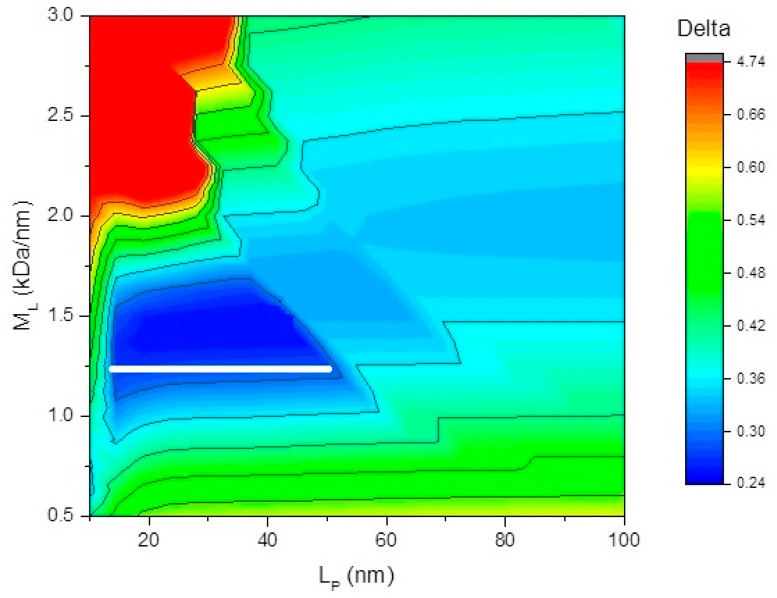
HYDFIT estimates of the persistence length, *L*_p_, based on the combined intrinsic viscosity, sedimentation coefficient, and molecular weight data of the three fractions. Because of the small number of data points, only a range of possible values could be specified, indicated by the white bar. The assumption is also made that the fractions have approximately the same conformation.

It is possible to combine the sets of viscosity-molecular weight values for each fraction with sedimentation-molecular weight values for each fraction to obtain an indication of conformational flexibility using the MULTI-HYDRO algorithm [26]: this algorithm is derived from a combination of the Bushin-Bohdanecky (viscosity) and Yamakawa-Fujita (sedimentation) relations. Figure 5 shows the results of the minimisation plot of the target function Δ (values shown in the panel on the right), which yields a value of *L*_p_ = 30 ± 20 nm. The large uncertainty is due to the (unavoidable) small number of data points (3 sets of intrinsic viscosity-sedimentation, coefficient-molecular weight data, corresponding to the 3 fractions, all of which are assumed to be part of a conformation series). Despite this limitation, the data appears consistent with a semi-flexible conformation, typical for pectic types of molecules [31]. For more precise conformation information, further data are required.

### 3.5. Complement Fixation

Inhibition of red blood cell lysis induced by the polysaccharide fractions was used to measure the ICH_50_ (which is the concentration of the polysaccharide samples required to inhibit 50% lysis) for all polysaccharide fractions to detect biological activity against human serum.

**Figure 6:**
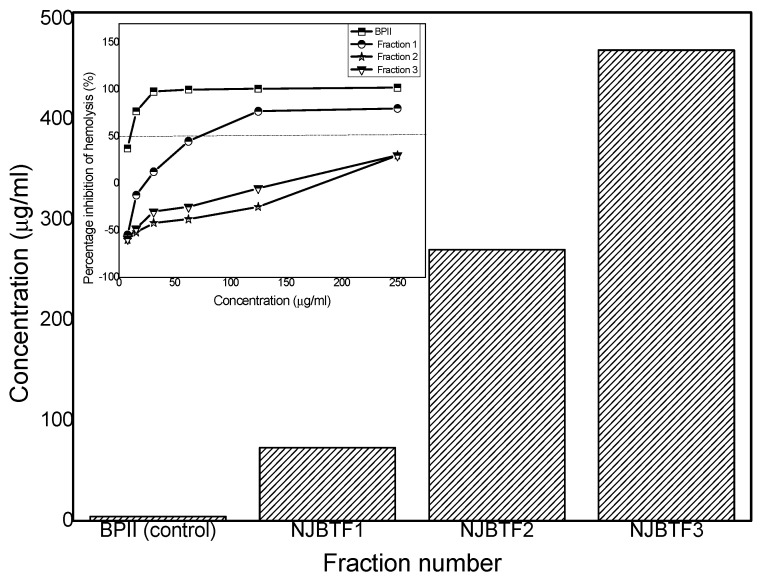
Dose-dependent activities of the *C. moschata* polysaccharide fractions (NJBTF1, 2, and 3) in the complement assay. BPII (*Biophytum petersianum*) was used as a positive control. Insert: includes the plot between percentage inhibition of hemolysis and concentration in µg for ICH_50_ calculation of BPII and NJBTF1, 2, and 3.

The ICH_50_ of the control, NJBTF1, F2, and F3 were 4.3, 73, 270, and 469 µg/mL, respectively, from the plot of the fraction number against the concentration (µg/mL) (Figure 6). It was observed that all fractions generated an immune response, and higher molecular weight fractions had higher bioactivity. Fraction 1, with the highest molecular weight (MW), was the most active sample, followed by fraction 2, and 3 with the least. These results suggest that the bioactivity of the cucurbit polysaccharide is linked to their molecular weight. Similar association of molecular weight of dextran polysaccharide and its immune response has been reported [40].

### 3.6. Monosaccharide Composition and Linkages

After extraction, fractionation, and collection of three respective fractions of the *C. moschata* polysaccharide, the monosaccharide composition of each fraction was determined using GC of the derived TMS-derivatives of the methylglycosides. This was followed by linkage analysis for the highest molecular weight fraction. The linkages were determined using GCMS on the alditols, which were partly acetylated and methylated. Monosaccharide analysis showed the major component of all fractions was an acidic monosaccharide, galacturonic acid (Table 4). NJBTF1 had glucuronic acid and a high proportion of neutral monosaccharides, including glucose and rhamnose.

In addition to galacturonic acid, NJBTF2 and NJBTF3 also contained neutral sugars. Glucuronic acid was not detected in these two fractions. The monosaccharide composition is in contrast with other studies performed on the unfractionated *C. moschata* polysaccharide [41,42]. The differences could be attributed to sensitivities in detection, as well as growing conditions and cultivation.

For linkage determination (Table 5), the higher molecular weight fraction (NJBTF1) was selected based on the observation that this fraction had the highest bioactivity. The linkages observed in the polysaccharide during this study contained (1→4) linked galacturonic acid and (1→2) linked rhamnose. The basic structure was pectin-like [43].

The alternating (1→4) linked galacturonic acid forms the smooth region or the linear backbone of the pectin and (1→2,4) linked rhamnose may form the hairy region of the pectin (type 1 rhamnogalacturonan). The presence of (1→3,6) linked galactose and arabinose suggests the presence of the arabinogalactan type II (AGII) side chain. These neutral sugars may be attached to RG-I as complex neutral side chains of arabinogalactans on position 4 of rhamnose and give rise to the so-called “hairy” region. A trace amount of xylose, another neutral sugar found in pectins [44,45], is also present.

Detection of a high amount of 2,6-Fru*f* and 1,2,6-Fru*f* indicated the presence of levan in the cucurbit with a highly branched structure (Table 5). It was not possible to identify the presence of fructans by GC using methanolysis. Methanolysis would have destroyed the fructan, and thus fructose could not have been detected with this method; only the glucose present in the fructan would be found. Methylation, on the other hand, does not have this limitation, hence the GCMS results revealing the undetected fructan and the type of linkages present.

Bacterial levan has been reported to elicit a bioactive response [46]: explicitly antidiabetic responses [47]. However, the presence of naturally occurring fructans in angiospermic plants is uncommon [48], and mostly only transgenic angiosperms contain fructans [49]. Nevertheless, in the present study, we have identified the presence of this rare non-pectin sugar along with pectin in the *C. moschata* polysaccharide. Hence, we propose that the presence of levan and pectin-like ingredients together could have contributed to the elevated level of bioactivity of NJBTF1 not seen to such a level in other fractions. In order to confirm the presence and proportion of levan in the total sample, further characterisation studies will be carried out using complementary methods such as NMR and FTIR in our laboratory.

## 4. Conclusions

The polysaccharide from *C. moschata* was extracted and analysed in unfractionated and fractionated samples.

Sedimentation velocity and equilibrium methods identified structural changes in the polysaccharide of *C. moschata* before and after fractionation. Changes observed in S, M, and [η] reflected variations in conformation upon fractionation. It is predicted that the polysaccharide from C*. moschata* has a semi-flexible coil conformation, although further data are necessary to confirm this prediction.

It was observed that the *C. moschata* polysaccharide was rich in galacturonic acid, rhamnose, and arabinose, as well as fructans. The highest molecular weight fraction (NJBTF1) also exhibited the highest bioactivity. Based on the immunogenic responses obtained as part of this study, it is anticipated that the polysaccharide obtained from *C. moschata* has the potential to be developed into a therapeutic agent.

It was revealed through GCMS analysis only that the levans (2,6-Fru*f* and 1,2,6-Fru*f*) are also present in NJBTF1 in addition to pectin. Our study confirms for the first time that levans, detected through GCMS, have been isolated from the pulp of cucurbit family. This finding offers a new horizon for levan-based phylogenetic research.

## Figures and Tables

**Table 1:** Intrinsic viscosity of *C. moschata* polysaccharide fractions.

	[η]_H_ (mL/g)	[η]_K_ (mL/g)	[η]_SC_ (mL/g)
NJBTF1	53.6 ± 1.3	53.6 ± 1.2	53.6 ± 1.3
NJBTF2	33.9 ± 0.6	34.0 ± 0.5	34.0 ± 0.6
NJBTF3	23.0 ± 0.7	23.0 ± 0.7	23.0 ± 0.7

± = standard error (and in other Tables below). [η]_H_: from Huggins extrapolation; [η]_K_: from Kramer extrapolation; [η]_SC_: from Solomon-Ciuta equation. Obtained at a temperature of 20.0 °C, in M buffer (pH = 7.0, I = 0.1).

**Table 2:** Sedimentation coefficients of *C. moschata* polysaccharide fractions.

	Peak 1		Peak 2		Peak 3		Weight Average
	s^o^_20,w_ (S)	wt frac (%)	s^o^_20,w_ (S)	wt frac (%)	s^o^_20,w_ (S)	wt frac (%)	s^o^_20,w_ (S)
**NJBTF1**	1.3 ± 0.1	81	3.5 ± 0.2	19	-	-	1.7 ± 0.2
**NJBTF2**	1.3 + 0.1	93	2.9 ± 0.6	5	4.1 ± 0.8	2	1.4 ± 0.2
**NJBTF3**	1.2 + 0.2	83	1.7 ± 0.7	17	-	-	1.3 ± 0.2

Samples in 0.1 M PBS, pH 7.0.

**Table 3:** Molecular weights of fractions of the *C. moschata* polysaccharide.

	*M*_w_ (kDa)	*M*_SM_ (kDa)
**NJBTF1**	90 ± 5	30 ± 4
**NJBTF2**	30 ± 2	20 ± 2
**NJBTF3**	19 ± 2	12 ± 2

Samples in 0.1 M PBS, pH 7.0. Weight average molecular weights, *M*_w_, from sedimentation equilibrium. Loading concentration is 0.4 mg/mL. Values extrapolated to c = 0, Scheraga-Mandelkern estimates *M*_SM_ (<*M*_w_ for polydisperse systems).

**Table 4:** Percentage of a SINGLE sugar related to the total amount of sugar obtained through gas chromatography. Tr = trace amount.

		NJBTF 1	NJBTF 2	NJBTF3
Neutral monosaccharides	Arabinose	3.6	4.4	2.8
	Rhamnose	11.7	4.8	2.8
	Fucose	tr	1.2	1.3
	Xylose	-	0.4	0.2
	Galactose	5.0	5.4	5.0
	Glucose	11.4	8.8	13.1
Acidic monosaccharides	Glucuronic acid	7.3	-	-
	Galacturonic acid	61.0	75.0	74.8

**Table 5:** Pectic and levan linkages obtained from GC-MS from the cucurbit polysaccharide fractions. Retention time is the elution time of the partly acetylated, partly methylated alditol acetates, obtained during the methylation process. “T” refers to the terminal monosaccharide.

Polymer	Retention Time (min)	Primary Fragments-Mass to Charge Ratio (m/z)	Identity of Origin	Relative Amount
PECTIN	12.99	45, 118, 161	T-Araf	3.0
	13.91	118, 131, 162, 175	T-Rha	1.8
	14.64	118, 131, 162, 175	T-Fuc	Tr.
	15.34	45, 190, 161	1,2 Araf	0.3
	15.40	45, 118, 233	1,3-Araf	0.5
	16.19	118, 189	1,5-Araf	Tr.
	16.23	131, 190,	1,2-Rha	7.7
	17.28	47, 118, 162, 163, 207	T-GlcA	1.0
	17.83	47, 118, 162, 163, 207	T-GalA	2.0
	18,37	131, 262	1,2,3-Rha	1.8
	18.47	118	1,3,5-Araf	0.4
	18.58	190, 203	1,2,4-Rha	2.0
	19.73	47, 118, 162, 235	1,4 GalA	65.9
	19.89	47, 118, 162, 235	1,4 GlcA	1.8
	20.13	45, 118, 161, 234, 277	1,3-Gal	3.0
	21.07	118, 162, 189, 233	1,6-Gal	0.3
	21.88	47, 190 235	1,2,4-GalA	0.8
	23.33	118, 189, 2234	1,3,6-Gal	2.3
LEVAN	16.16; 16.32	45, 161, 162	T-Fruf	18
	17.28	45, 118, 161, 162, 205	T-Glc	1
	19.37; 19.55	45, 162, 189	2,6-Fruf	245
	22.61	189, 190	1,2,6-Fruf	16

## Abbreviations

C	(g/mL or Rayleigh fringe units), concentration
ICH_50_	concentration of the polysaccharide samples required to inhibit 50% lysis
*L*_p_	persistence length (measure of flexibility) (cm)
*M*_w,app,_	apparent weight-average molecular weight (Da)
*M*_w_	weight average molecular weight (Da)
*M*_SM_	Scheraga-Mandelkern molecular weight (Da)
*M*_L_	mass per unit length (Da. cm^−1^)
NJBTF1	fraction 1 (high molecular weight fraction)
NJBTF2	fraction 2 (medium molecular weight fraction)
NJBTF3	fraction 3 (low molecular weight fraction)
H	viscosity (Poise)
[η]	intrinsic viscosity (ml/g)
ῡ	partial specific volume
*s*_20,w_	weight average sedimentation coefficient, normalised to standard solvent conditions (density and viscosity of water at 20.0 °C)
*s*^o^_20,w_, *s*_20,w_	corrected for non-ideality (extrapolation to *c* = 0)

## References

[1] Niture S.K., Refai L. (2013). Plant pectin: A potential source for cancer suppression. Am. J. Pharmacol. Toxicol..

[2] Dutta P.K., Tripathi S., Mehrotra G.K., Dutta J. (2009). Perspectives for chitosan based antimicrobial films in food applications. Food Chem..

[3] Espitia P.J.P., Wu W.X., Avena-Bustillos R.J., Soares N.F., McHugh T.H. (2014). Edible films from pectin: Physical-mechanical and antimicrobial properties-A review. Food Hydrocoll..

[4] Beneke C.E., Viljoen A.M., Hamman J.H. (2009). Polymeric plant-derived excipients in drug delivery. Molecules.

[5] Huang H.J., Yuan W.K., Chen X.D. (2006). Microencapsulation Based on Emulsification for Producing Pharmaceutical Products: A Literature Review. Dev. Chem. Eng. Miner. Process..

[6] Villanova J.C.O., Ayres E., Oréfice R.L. (2015). Design, characterization and preliminary in vitro evaluation of a mucoadhesive polymer based on modified pectin and acrylic monomers with potential use as a pharmaceutical excipient. Carbohydr. Polym..

[7] Hsieh Y.S.Y., Liao S.F., Yang W.B. (2009). Biologically Active Polysaccharides in Medicinal Plants. N. Z. J. For. Sci..

[8] Giosafatto C.V.L., Mariniello L., Ring S. (2007). Extraction and Characterization of Foeniculum vulgare Pectins and Their Use for Preparing Biopolymer Films in the Presence of Phaseolin Protein. J. Agric. Food Chem..

[9] Fissore E.N., Ponce N.M., Stortz C.A., Rojas A.M., Gerschenson L.N. (2007). Characterisation of Fiber Obtained from Pumpkin (cucumis moschata duch.) Mesocarp Through Enzymatic Treatment. Food Sci. Technol. Int..

[10] Guillon F., Champ M. (2000). Structural And Physical Properties of Dietary Fibres, and Consequences of Processing on Human Physiology. Food Res. Int..

[11] Zhang W., Xu P., Zhang H. (2015). Pectin in cancer therapy: A review. Trends Food Sci. Technol..

[12] Grønhaug T.E., Ghildyal P., Barsett H., Michaelsen T.E., Morris G., Diallo D., Inngjerdingen M., Paulsen B.S. (2010). Bioactive Arabinogalactans from the Leaves of OPILIA Celtidifolia Endl. ex Walp. (Opiliaceae). Glycobiology.

[13] Jacobo V.N., Maróstica M.R., Zazueta J.J., Gallegos J.A. (2011). Physicochemical, technological properties, and health-benefits of Cucurbita moschata Duchense vs. Cehualca: A Review. Food Res. Int..

[14] Asgary S., Moshtaghian S.J., Setorki M., Kazemi S., Rafieian-Kopaei M., Adelnia A., Shamsi F. (2011). Hypoglycaemic and Hypolipidemic Effects of Pumpkin (Cucurbita pepo L.) on Alloxan-Induced Diabetic Rats. Afr. J. Pharm. Pharmacol..

[15] Nosáľová G., Prisenžňáková Ľ., Košťálová Z., Ebringerová A., Hromádková Z. (2011). Suppressive effect of pectic polysaccharides from Cucurbita pepo L. var. Styriaca on citric acid-induced cough reflex in guinea pigs. Fitoterapia.

[16] Košťálová Z., Hromádková Z., Ebringerová A. (2009). Chemical evaluation of seeded fruit biomass of oil pumpkin (Cucurbita pepo L. var. Styriaca). Chem. Pap..

[17] Quanhong L., Caili F., Yukui R., Guanghui H., Tongyi C. (2005). Effects of Protein-Bound Polysaccharide Isolated from Pumpkin on Insulin in Diabetic Rats. Plant Foods Hum. Nutr..

[18] Li X., Zhao R., Zhou H.L., Wu D.H. (2012). Deproteinization of Polysaccharide from the Stigma Maydis by Sevag Method. Adv. Mater. Res..

[19] Sam K.C.C., Neilson S. (2003). Compositional analysis of foods. Food Analysis.

[20] Theisen A., Johann C., Deacon M.P., Harding S.E. (2000). Refractive Increment Data-Book for Polymer and Biomolecular Scientists.

[21] Harding S.E. (1997). The intrinsic viscosity of Biological Macromolecules. Progress in Measurement, Interaction and Application to Structure in Dilute Solution. Prog. Biophys. Mol. Biol..

[22] Schuck P. (2000). Size-distribution analysis of macromolecules by sedimentation velocity ultracentrifugation and lamm equation modeling. Biophys. J..

[23] Schuck P., Harding S.E., Gillis R.B., Besong T.M.D., Almutairi F., Adams G.G., Rowe A.J. (2014). SEDFIT-MSTAR: Molecular weight and molecular weight distribution analysis of polymers by sedimentation equilibrium in the ultracentrifuge. Analyst.

[24] Scheraga H.A., Mandelkern L. (1953). Consideratioi of the hydrodynamic properties of proteins. J. Am. Chem. Soc..

[25] Ortega A. (2005). Metodologías Computacionales Para Propiedades en Disolución de Macromoléculas Rígidas y Flexibles. Ph.D. Thesis.

[26] Ortega A., Garcı´a de la Torre J. (2007). Equivalent radii and ratios of radii from solution properties as indicators of macromolecular conformation, shape, and flexibility. Biomacromolecules.

[27] Bushin S., Tsvetkov V., Lysenko E., Emelianov V. (1981). The sedimentation diffusion and viscometric analysis of the conformation properties and molecular rigidity of ladder-like polyphenyl siloxane in solution. Vysok. Soedin.

[28] Bohdanecky M. (1983). New method for estimating the parameters of the wormlike chain model from the intrinsic viscosity of stiff-chain polymers. Macromolecules.

[29] Yamakawa H., Fujii M. (1973). Translational friction coefficient of wormlike chains. Macromolecules.

[30] Kök M.S., Abdelhameed A.S., Ang S., Morris G.A., Harding S.E. (2009). A novel global hydrodynamic analysis of the molecular flexibility of the dietary fibre polysaccharide konjac glucomannan. Food Hydrocoll..

[31] Morris G.A., Garcia de la Torre J., Ortega A., Castile J., Smith A., Harding S.E. (2008). Molecular flexibility of citrus pectins by combined sedimentation and viscosity analysis. Food Hydrocoll..

[32] Michaelsen T.E., Gilje A., Samuelsen A.B., Høgåsen K., Paulsen B.S. (2000). Interaction Between Human Complement and a Pectin Type Polysaccharide Fraction, PMII, from the Leaves of *Plantago major* L.. Scand. J. Immunol..

[33] Chambers R.E., Clamp J.R. (1971). An assessment of methanolysis and other factors used in the analysis of carbohydrate-containing materials. Biochem. J..

[34] Sims I.M., Bacic A. (1995). Extracellular polysaccharides from suspension cultures of Nicotiana plumbaginifolia. Phytochemistry.

[35] Fissore E.N., Ponce N.M., de Escalada Pla M., Stortz C.A., Rojas A.M., Gerschenson L.N. (2010). Characterization of Acid-Extracted Pectin-Enriched Products Obtained from Red Beet (Beta vulgaris L. var. conditiva) and Butternut (Cucurbita moschata Duch ex Poiret). J. Agric.Food Chem..

[36] Roura S.I., Valle C.E.D., Aguero L., Davidovich L.A. (2007). Changes in Apparent Viscosity and Vitamin C Retention During Thermal Treatment Of Butternut Squash (Cucurbita Moschata Duch) Pulp: Effect of Ripening Stage. J. Food Qual..

[37] Harding S.E. (2005). Challenges for the modern analytical ultracentrifuge analysis of polysaccharides. Carbohydr. Res..

[38] Dam J., Schuck P. (2004). Calculating sedimentation coefficient distributions by direct modeling of sedimentation velocity concentration profiles. Methods Enzymol..

[39] Creeth J.M., Pain R.H. (1967). The determination of molecular weights of biological macromolecules by ultracentrifuge methods. Prog. Biophys. Mol. Biol..

[40] Kabat E.A., Bezer A.E. (1958). The effect of variation in molecular weight on the antigenicity of dextran in man. Arch. Biochem. Biophys..

[41] Du B., Song Y., Hu X., Liao X., Ni Y., Li Q. (2011). Oligosaccharides prepared by acid hydrolysis of polysaccharides from pumpkin (Cucurbita moschata) pulp and their prebiotic activities. Int. J. Food Sci. Technol..

[42] Yang X., Zhao Y., Lv Y. (2007). Chemical Composition and Antioxidant Activity of an Acidic Polysaccharide Extracted from Cucurbita moschata Duchesne ex Poiret. J. Agric. Food Chem..

[43] Izydorczyk M., Cui S.W., Wang Q., Cui S.W. (2005). Polysaccharide gums: Structures, functional properties, and applications. Food Carbohydrates: Chemistry, Physical Properties, and Applications.

[44] Voragen A.G.J., Coenen G.J., Verhoef R.P., Schols H.A. (2009). Pectin, a versatile polysaccharide present in plant cell walls. Struct. Chem..

[45] Wang J., Zhang Y., Luo J. (2018). Structure elucidation of a pectin from Dendrobium nobile Lindl and its immunological activity. Biotechnol. Biotechno. Equip..

[46] Öner E.T., Hernández L., Combie J. (2016). Review of Levan polysaccharide: From a century of past experiences to future prospects. Biotechnol. Adv..

[47] Dahech I., Belghith K.S., Hamden K., Feki A., Belghith H., Mejdoub H. (2011). Oral administration of levan polysaccharide reduces the alloxan-induced oxidative stress in rats. Int. J. Biol. Macromol..

[48] Aspinall G.O., Percival E., Rees D.A., Rennie M., Coffey S. (1967). Oligosaccharides, Polysaccharides and Related Compounds. Rodd’s Chemistry of CarbonCompounds.

[49] Banguela A., Arrieta J.G., Rodríguez R., Trujillo L.E., Menéndez C., Hernández L. (2011). High levan accumulation in transgenic tobacco plants expressing the Gluconacetobacter diazotrophicus levansucrase gene. J. Biotechnol..

